# Metastatic Thyroid Osteosarcoma With Concomitant Multifocal Papillary Carcinoma Presenting as a Collision Tumor

**DOI:** 10.7759/cureus.15425

**Published:** 2021-06-03

**Authors:** Nektarios Koufopoulos, Andriani Zacharatou, Alina-Roxani Gouloumis, Nikolaos Papadimitriou, Periklis Tomos, Periklis G Foukas, Ioannis G Panayiotides

**Affiliations:** 1 2nd Department of Pathology, National and Kapodistrian University of Athens, “Attikon” University Hospital, Athens, GRC; 2 2nd Department of Ear, Nose, Throat (ENT), National and Kapodistrian University of Athens, “Attikon” University Hospital, Athens, GRC; 3 Department of Thoracic Surgery, National and Kapodistrian University of Athens, “Attikon” University Hospital, Athens, GRC

**Keywords:** osteosarcoma, metastasis, thyroid, papillary carcinoma, collision tumor

## Abstract

Metastatic involvement of the thyroid occurs rarely, by either hematogenous spread or direct extension from adjacent organs. The most frequent metastatic tumors are clear cell, renal cell, lung, breast, and squamous cell carcinoma.

The occurrence of osteosarcoma and papillary thyroid carcinoma in the same patient is rare, with only a few reported cases in the literature. On the other hand, only one case of osteosarcoma thyroid metastasis has so far been reported.

We herewith present another case with metastatic osteosarcoma and multifocal papillary thyroid carcinoma presenting as a collision tumor and review the relevant literature.

## Introduction

Metastases to the thyroid constitute only 2%-3% of all malignant thyroid tumors [1]. Carcinomas of the kidney (clear cell renal cell carcinoma), lung, breast, as well as squamous cell carcinomas (head and neck, esophageal, and lung), are the most frequent malignancies reported so far. Other types, such as carcinomas of the gastrointestinal tract or malignant melanoma, are less frequent [2]. Secondary involvement of the thyroid may occur either by the direct extension of neoplasms arising in adjacent organs (squamous cell carcinoma, parathyroid carcinoma, soft tissue malignancies) or by hematogenous spread. The relative rarity of hematogenous metastases to the thyroid despite its rich vascularization has been explained by two major hypotheses: 1) rapid blood flow does not facilitate the adhesion and seeding of metastases; 2) growth of malignant cells may be inhibited by high oxygen saturation and iodine content [3].

Osteosarcoma is one of the rarest metastatic tumors to the thyroid, with only one case having been reported so far, to our knowledge [4].

A collision tumor occurs when two different tumor types are located within the same anatomic location. These tumors may be benign, malignant, or, less commonly, metastatic, occurring in any combination [5- 6].

We herewith report a case of osteosarcoma metastasis to the thyroid with the concomitant presence of multifocal papillary thyroid carcinoma presenting as a collision tumor.

## Case presentation

**Figure 1:**
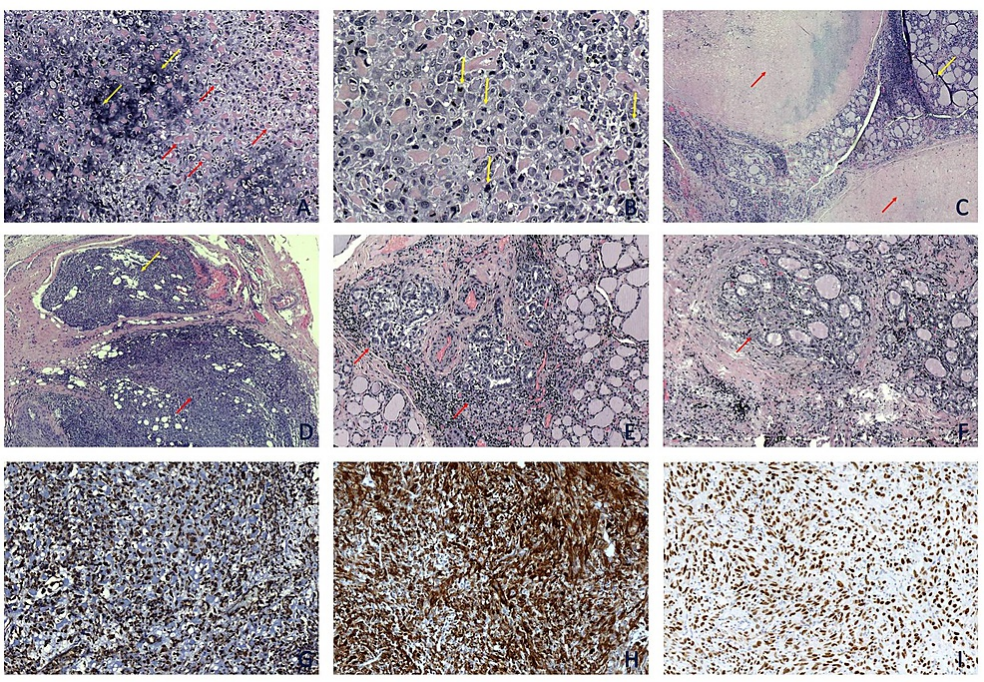
Immunohistochemical study of the sarcomatous tumor A: Malignant tumor with cartilage (yellow arrows) and osteoid production (red arrows) (H&E, x10). B: Pleomorphic tumor cells with many mitoses (yellow arrows) (H&E, x40). C: Two necrotic and fibrotic foci (red arrows) within thyroid parenchyma (yellow arrow) (H&E, x4). D: Osteosarcoma (red arrow) adjacent to a normal parathyroid (yellow arrow) (H&E, x4). E & F: Microscopic foci of follicular type papillary thyroid carcinoma (red arrow) surrounded by normal thyroid parenchyma (yellow arrow) (H&E, x10). G & H & I: The immunohistochemical study was positive for Vimentin (G), SMA (H), and SATB2 (I) (Vim, SMA, and SATB2, x 10).

A 60-year-old female presented with a palpable thyroid mass. Past history was significant for a chondroblastic osteosarcoma of the sacrum operated elsewhere nine years ago; metastasis to the upper lobe of the left lung with a maximum diameter of 6 cm was excised four years later in our hospital. The patient received no further treatment. Fine-needle aspiration cytology of the thyroid mass has been reported as consistent with papillary thyroid neoplasm (Bethesda class VI). Consequently, a total thyroidectomy was performed. The left thyroid lobe was almost totally replaced by a well-circumscribed, solid, gray-whitish, hard, 6.2 cm large tumor while the right lobe was enlarged diffusely and symmetrically. On microscopic examination, the tumor consisted of a sarcomatous neoplasm with high-grade nuclear atypia, pleomorphism, several mitotic figures, extensive necrosis, and focal fibrosis. Several areas of high-grade cartilage and osteoid production were recognized (Figures 1A-1C). The tumor infiltrated the perithyroidal fat and skeletal muscle, abutting to a normal parathyroid gland (Figure 1D). Moreover, three, microscopic (1 - 1.5 mm large) foci of follicular type papillary thyroid carcinoma were found in the same lobe, all within thyroid parenchyma (Figure 1E). Three lymph nodes were found contiguous to the thyroid; two (including the Delphian), measuring 1 - 5 mm, were infiltrated by papillary thyroid carcinoma without extracapsular extension. The remaining thyroid parenchyma of the left lobe, as well as the right lobe, showed evidence of Hashimoto thyroiditis. An immunohistochemical study of the sarcomatous tumor showed positive staining for Vimentin (Figure 1F), SMA (Figure 1G), and SATB2 (Figure 1H). Tumor cells did not express CKAE1/AE3, CK8/18, EMA, CK14, or CK7.

For diagnostic purposes, we reviewed the slides of the metastasis to the lung that were available since the diagnosis of the primary tumor was done at another hospital.

**Figure 2:**
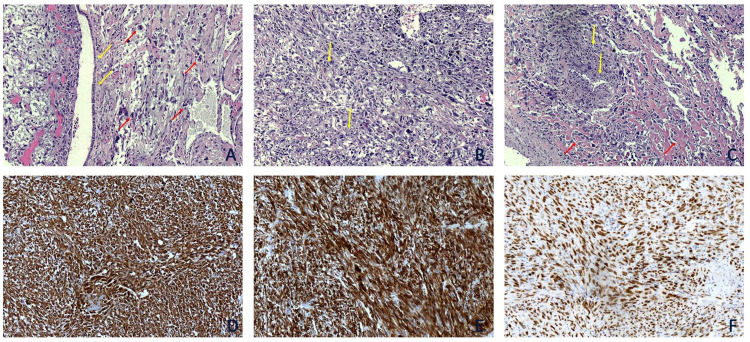
Slides of the metastasis to the lung A: Tumor cells showing pleomorphism and several mitotic figures (red arrows) adjacent to the bronchial epithelium (yellow arrows) (H&E, x10). B & C: The tumor had several areas with chondromyxoid (yellow arrows) and focal osteoid production (red arrows) (H&E, x10). D & E & F: Immunohistochemically, tumor cells uniformly expressed Vimentin (D), SMA (E) and SATB2 (F) (Vimentin, SMA, and SATB2 x10).

Tumor cells were pleomorphic (Figure 2A) and several mitoses were readily identifiable (Figure 2B). Areas of atypical cells embedded in the chondromyxoid matrix and focal osteoid production were recognized (Figure 2C). Immunohistochemically, tumor cells expressed Vimentin (Figure 2D), SMA (Figure 2E), and SATB2 (Figure 2F). Focal faint staining for CD56 was observed. Markers of epithelial (CKAE1/AE3, CK8/18, EMA, CK14, CK7) and melanocytic (HMB45, Melan A, MITF, Tyrosinase) differentiation were negative.

A diagnosis of metastasis of the previously diagnosed osteosarcoma to the left thyroid lobe, with a coexisting multifocal papillary thyroid carcinoma, was rendered. The patient's recovery was uneventful; she was released seven days after surgery. No further treatment was given. The patient died a year later due to widespread metastatic osteosarcoma; permission for autopsy was not granted.

## Discussion

Metastases to the thyroid are an infrequent occurrence. Clear cell renal cell carcinoma is the most frequently occurring secondary, followed by lung and breast carcinomas.

Patients with thyroid metastases usually have a history of previously diagnosed malignancy, whereas the thyroid tumor is the first manifestation in 20%-40% of cases [3,7]. Presenting symptoms usually closely mimic primary thyroid tumors; less often, a metastasis may give a clinical clue to the primary malignancy [8]. Imaging studies (computed tomography scan, magnetic resonance imaging, Positron emission tomography-computed tomography (PET-CT)) cannot reliably distinguish between primary and metastatic thyroid tumors [9].

Thyroid fine-needle aspiration cytology, combined with the patient's history and other ancillary studies, may offer a rapid and accurate diagnostic tool. However, a thyroid secondary may sometimes simulate a primary thyroid neoplasm even on cytology [2]. Thus, pathological examination of the surgical specimen remains, in most cases, the only reliable diagnosis of thyroid metastasis.

The histologic differential diagnosis of metastatic osteosarcoma in the first place includes primary osteosarcoma of the thyroid and anaplastic carcinoma of the thyroid with extensive osseous metaplasia; medullary thyroid carcinoma, sarcomatoid poorly differentiated thyroid carcinoma, spindle epithelial tumor with thymus-like differentiation, and synovial sarcoma [10] may also be considered in case of lack of osteoid production. In our case, a history of a twice previously diagnosed osteosarcoma (both in its primary and in a pulmonary metastasis), the similar histological and immunohistochemical findings of the metastatic lung and thyroid tumors, and the lack of histological continuity of the three foci of papillary thyroid carcinoma with the sarcomatous tumor (excluding the diagnosis of anaplastic thyroid carcinoma with osteosarcomatous differentiation) were essential in establishing the correct diagnosis. This is of utmost importance in order to choose the appropriate treatment [2]. Treatment of thyroid secondaries mainly aims at palliation. Occasionally, surgery may be performed to achieve local control or long-term cure in selected patients [1,8].

Concerning the coexistence of osteosarcoma with primary thyroid carcinoma, it is noteworthy that they both may arise in the context of two genetic syndromes: multiple hamartoma syndrome (Cowden disease) and Werner syndrome [11-12]. No clinical signs of either syndrome were present in our patient.

The incidence of second primary cancers after osteosarcoma treatment is not very high [13]. We conducted a search on PubMed using the terms “osteosarcoma AND papillary thyroid carcinoma,” “osteosarcoma metastasis AND thyroid,” and “thyroid metastasis AND osteosarcoma.” Our search yielded 11 cases of coexisting osteosarcoma and papillary thyroid carcinoma in the same patient [11-12,14-18]. Papillary thyroid carcinoma occurred after osteosarcoma in seven cases [11,14-16], whereas in two cases, osteosarcoma presented as a second or third malignancy [12]. The case reported by Tsuchiya et al. concerned the occurrence of five different primary tumors (i.e., osteosarcoma of the left distal tibia, malignant melanoma of the left plantar region, gastric carcinoma, leiomyosarcoma of the lung and papillary thyroid carcinoma) in the same patient, within a period of six months [17]. Finally, Dannenberg et al. reported a case of simultaneous osteosarcoma and papillary thyroid carcinoma in a 15-year-old patient [18]. The clinical features of these cases are summarized in Table 1.

**Table 1:** Clinical data of patients with papillary thyroid carcinoma and osteosarcoma Ca: carcinoma; M: male; F: female

Case nr.	Authors	Year	Age	Sex	1st malignancy	2nd malignancy	3rd malignancy	Comments
1	Tsuchiya et al. [17]	1991	45	M	Osteosarcoma, thyroid Ca, melanoma, gastric Ca, leiomyosarcoma			Werner syndrome
2	Yen et al. [11]	1993			Thyroid Ca	Osteosarcoma	Breast in situ Ca	Cowden disease
3	Jimenez et al. [15]	1995	13, 21	M	Osteosarcoma	Thyroid Ca		
4	Goto et al. [12]	1996	27,34,35	F	Thyroid Ca	Uterus (no further specification)	Osteosarcoma	Werner syndrome
5	Goto et al. [12]	1996	40, 49	F	Thyroid Ca	Osteosarcoma	-	Werner syndrome
6	Verneris et al. [14]	2001	14, 30	M	Osteosarcoma	Thyroid Ca		
7	Verneris et al. [14]	2001	13, 22	F	Osteosarcoma	Thyroid Ca		
8	Verneris et al. [14]	2001		M	Osteosarcoma	Thyroid Ca		
9	Dannenberg et al. [18]	2005	15	F	Osteosarcoma, thyroid Ca			
10	Kim et al. [16]	2008	13, 25	F	Osteosarcoma	Thyroid Ca		
11	Kim et al. [16]	2008	18, 23	M	Osteosarcoma	Thyroid Ca		

## Conclusions

Thyroid secondaries, however rare, should always be included in the differential diagnosis, in the presence of relevant patient history. Although imaging studies and fine-needle aspiration cytology may be suggestive, histopathologic examination of the surgical specimen remains the final diagnosis.
